# Nanoscale Characterization of Surface Plasmon-Coupled Photoluminescence Enhancement in Pseudo Micro Blue LEDs Using Near-Field Scanning Optical Microscopy

**DOI:** 10.3390/nano10040751

**Published:** 2020-04-15

**Authors:** Yufeng Li, Aixing Li, Ye Zhang, Peng Hu, Wei Du, Xilin Su, Qiang Li, Feng Yun

**Affiliations:** 1 Shaanxi Provincial Key Laboratory of Photonics & Information Technology, Xi’an Jiaotong University, Xi’an 710049, China; yufengli@mail.xjtu.edu.cn (Y.L.); liaixing@stu.xjtu.edu.cn (A.L.); zhangye830108@mail.xjtu.edu.cn (Y.Z.); 13679135338@163.com (P.H.); duweipersonal@163.com (W.D.); suxilin111@163.com (X.S.); liqiang_ynu@126.com (Q.L.); 2 Solid-State Lighting Engineering Research Center, Xi’an Jiaotong University, Xi’an 710049, China

**Keywords:** micro-LEDs, localization surface plasmon, near-filed scanning optical microscopy

## Abstract

The microcave array with extreme large aspect ratio was fabricated on the p-GaN capping layer followed by Ag nanoparticles preparation. The coupling distance between the dual-wavelength InGaN/GaN multiple quantum wells and the localized surface plasmon resonance was carefully characterized in nanometer scale by scanning near-field optical microscopy. The effects of coupling distance and excitation power on the enhancement of photoluminescence were investigated. The penetration depth was measured in the range of 39–55 nm depending on the excitation density. At low excitation power density, the maximum enhancement of 103 was achieved at the optimum coupling distance of 25 nm. Time-resolved photoluminescence shows that the recombination life time was shortened from 5.86 to 1.47 ns by the introduction of Ag nanoparticle plasmon resonance.

## 1. Introduction

Micro-emitters are widely used in micro displays, mask-free photolithography, highly parallel on-chip fluorescence detection and other fields. Despite efficient micro-blue and white light emitting diodes (LEDs) based on GaN/InGaN multi-quantum-wells (MQWs), the plasmonic effect integrated with metallic nanostructures as one promising method to further improve the opto-electrical performance of micro-LEDs still gains enormous interest [1,2]. Plasmonic micro-LEDs have recently become the forefront of research and development aimed at the creation of new extremely efficient micro emitters for the visible-ultraviolet (UV) spectral range and visible light communication [3,4]. The localized surface plasmons (LSPs) can efficiently and rapidly oscillate with exciton energy in MQWs [5,6,7,8,9,10,11,12,13,14,15], which causes changes of polarization and internal quantum efficiency [16,17]. However, most of the reported studies above focused on the macroscopic enhancement using micro-photoluminescence (PL) mapping [12]. Given the small size, it is difficult to experimentally evaluate the SP effect in high spatial resolution of a micro-LED. The LSPs coupling with the exciton in MQWs through its extended electro-magnetic field can occur in the near-field range and the effective transfer of energy between excitons in MQWs and surface plasmons (SPs) has a limited distance of approximately several tens of nanometers [4,13,18]. The SP is an evanescent wave that exponentially decays with distance from the metal surface. The penetration depth *Z* of the SP fringing electrical field into the semiconductor is given by *Z* = λ/2π[(ε′_GaN_ – ε′_metal_)/ε′_metal_^2^]^1/2^ where ε′_GaN_ and ε′_metal_ are the real parts of the dielectric constants of the semiconductor and metal and λ is the wavelength of the SP resonance. For example, the penetration depth of the SP fringing field into the semiconductor was calculated as 47 nm for Ag [6]. Due to the limitation of spatial resolution, the optimal vertical SP coupling distance, defined as the physical gap distance between the LSP and the MQWs, was mostly estimated by simulation or calculation. A more accurate experimental measurement is desired to investigate the optimal coupling distance and penetration depth of the SP field. In this work, we experimentally demonstrate a novel plasmonic LED configuration, incorporating microcave arrays structures with extremely large aspect ratio through the p-GaN layer covered by distributed Ag nanoparticles (NPs). The nanoscale characterization of near-filed coupled PL enhancement was studied by near-filed scanning optical microscopy (NSOM) techniques. The transverse dimension of the microcave is similar to that of a typical micro-LED with pixel diameters of 30–50 µm. Large aspect ratio enables us to measure the coupling distance dependent LSP effect with high accuracy.

## 2. Materials and Methods

GaN-based LED epi-layers were grown on double-side polished c-oriented patterned sapphire substrate (PSS) by metal-organic vapor phase epitaxy (MOVPE). 20 nm low temperature (LT)-GaN buffer layer was deposited on the substrate. After annealing, 2.5 µm un-doped GaN layer and 2.5 µm Si-doped (1 × 10^19^/cm^3^) GaN layer were grown. The active region of the blue LED consists of 3 pairs of GaN/In_0.11_Ga_0.89_N bottom MQWs-2 with emission wavelength at 417 nm followed by 5 pairs of GaN/In_0.16_Ga_0.84_N top MQWs-1 with emission wavelength at 445 nm. It was then covered by 30 nm p-Al_0.27_Ga_0.73_N:Mg electron blocking layer (EBL), and 70 nm p-GaN:Mg p-contact layer. 2.9 μm positive photoresist (WH-390PG) was spun on the wafer, and a cylindrical holes array structure with a diameter of 70 μm and a depth of 2.9 μm was produced by photolithography technology. The photoresist was reflowed at 160 °C for 30 min. Then, the second layer of photoresist was deposited and reflowed at 180 °C for 40 min. The mask pattern of the photoresist was transformed from hole array to a microcave array. P-GaN layer was etched by inductively coupled plasma (ICP) dry etching to form uniform microcave structure. Sample 1 and 2 were etched for 175 nm with an opening diameter of 46 μm and sample 3 and 4 were etched for 75 nm with an opening diameter of 32 μm. Samples were immersed into 3 mol/L KOH solution at 50 °C for 30 min after photoresist striping to reduce the surface roughness. Subsequently, 6 nm Ag film was deposited on top of the etched p-GaN layer of sample 2 and 4 using thermal evaporation and both samples were annealed in N_2_ atmosphere at 300 °C for 3–5 min. The Ag film was transformed into random NPs after thermal annealing. For comparison, we also prepared microcave array LED without Ag NPs (sample 1 and 3). The size and density of Ag NPs on top of the LED surfaces were measured by scanning electron microscope (SEM) (FEI, Hillsboro, OR, USA). The near-field PL measurements were performed using NSOM ((NTEGRA, NT-MDT, Moscow, Russia) with a tip aperture size of 100 nm in illumination-mode. The 405 nm continuous wave (CW) laser was introduced to the NSOM system and focused onto the p-GaN surface with a spot size of 150 nm. Such small aperture enables us to get optical excitation power density as high as 3.6 MW/cm^2^. The PL intensity and the spectra were measured by a 100× objective lens through the polished backside of the sapphire substrate. A 409-nm long-bandpass filter was installed in the detector system to eliminate the excitation laser signal. The spectra were recorded by a spectrometer (Horibai-HR550, Jobin Yvon, Kyoto, Japan) with a resolution of 0.02 nm. Time-resolved PL (TRPL) measurements were performed using a FLS980 fluorescence spectrofluorometer and a standard distribution refrigeration red-sensitive photon count photomultiplier tube (R928P) with a minimum temporal resolution of 305 fs/channel. The excitation source was EPL-375 nm laser (Edinburgh Instruments Ltd., Edinburgh, England) with maximum average power of 5 mW. The temperature dependent PL was measured from 2–300 K to calculate the internal quantum efficiency (IQE).

## 3. Results and Discussion

**Figure 1:**
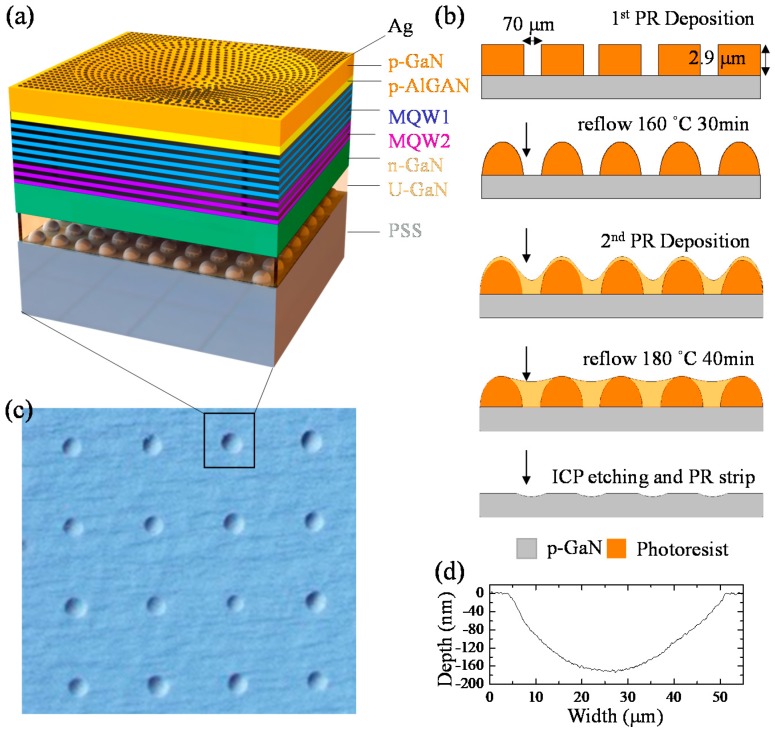
(**a**) LSP-LEDs structure diagram. (**b**) Fabrication process of microcave array structure. (**c**) Top-view optical image of the microcave array structure. (**d**) The depth profile of a single microcave.

Figure 1a shows the schematic diagram of the LSP-LED. The fabrication process of microcave array structure is shown in Figure 1b. A microcave array with a curvature of 5.56 × 10^−4^ μm^−1^, an outer diameter of 32–46 μm and aspect-ratio of more than 400:1 was obtained (Figure 1c). Such a large aspect ratio was chosen to minimize the change of light extraction efficiency (LEE) caused by the curved shape of the microcave. The absorption coefficient of p-GaN at 405 nm (excitation) and 445 nm (emission) are about 100 and 20 cm^−1^ respectively [19]. This is equivalent to the change of LEE about 1 × 10^−^^3^. Therefore, the total LEE variation caused by the difference of surface curvature and thickness is small enough to be ignored and the only enhancement obtained via QW-SP coupling can be separated [9]. Figure 1d shows the depth profile of a single microcave. Before silver deposition, the surface morphology of the microcave was reduced to 3.7 nm (measured by Atomic Force Microscope) by KOH etching, further reducing the LEE change caused by the surface roughness.

**Figure 2:**
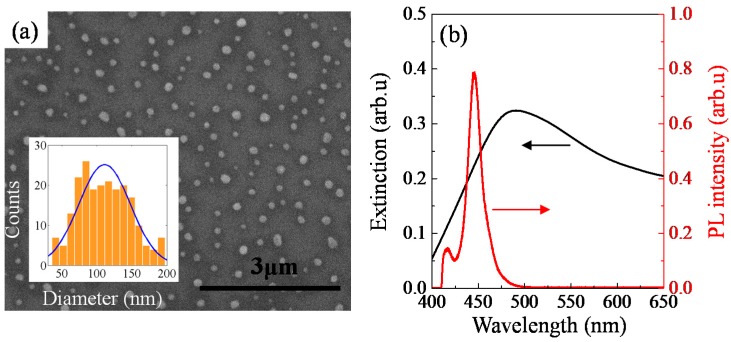
(**a**) The plane-view SEM image of Ag NPs inside the microcave, inset: diameter distribution of Ag NPs and (**b**) Extinction spectrum of GaN template with Ag NPs and PL spectrum of MQWs.

The plane-view secondary electron microscope (SEM) image of a microcave with Ag NPs is shown in Figure 2a. The diameter distribution of the Ag NPs is shown in the inset of Figure 2a. The median diameter of NPs is 110 nm and the density of the NPs is calculated as 4.46 × 10^8^ cm^−2^.

The center of surface-plasmon resonance is highly dependent on the density and size of Ag NPs on the surface [20]. The extinction peak was attributed to the dipolar resonances in the NPs. By changing the duration time of annealing, the geometrical properties and the density of the Ag NPs were changed. As a result, the peak of the extinction curve shifts from 510 nm (3 min annealing) to 490 nm (5 min annealing). Figure 2b shows the extinction curve of the Ag NPs annealed for 5 min. From the wide spread of the size distribution of Ag NPs one can anticipate a certain broadening of the extinction curve. The large full width at half maximum (FWHM) was due to different size and spacing of Ag NPs which leads to the inhomogeneous broadening of LSP resonances [21]. The main PL emission peaks from MQWs-1 is located at 445 nm with a FWHM of 16.9 nm, which shows a relatively good overlap with the extinction curve. The secondary peak with much smaller PL amplitude from the MQWs-2 is located at 417 nm with a FWHM of 10.9 nm and the overlap between MQWs-2 and extinction curve is small.

**Figure 3:**
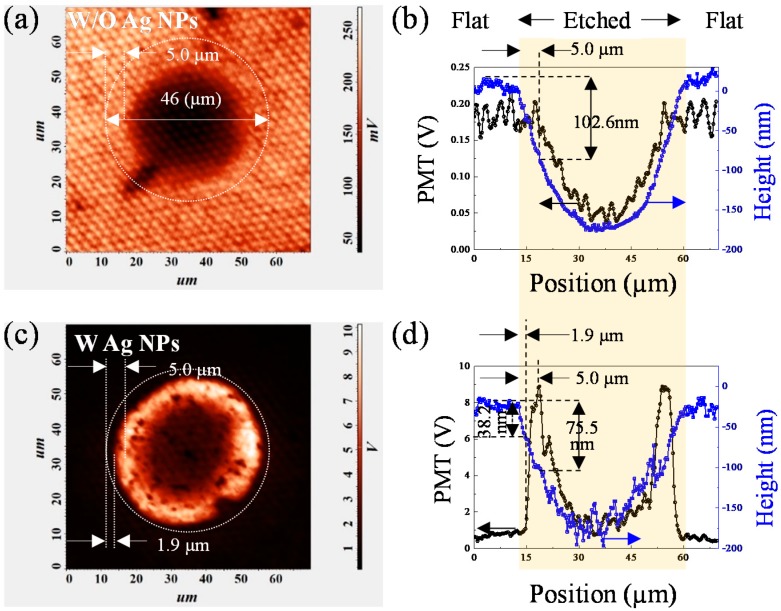
(**a**) Near-field PL mapping and (**b**) cross sectional PL profile and height profile through the center of microcave of sample 1 without Ag NPs; (**c**) Near-field PL mapping and (**d**) PL and height profiles of the microcave of sample 2 with Ag NPs.

Figure 3 shows PL intensity mapping at 1.3 MW/cm^2^ and cross-sectional depth profile for sample 1 and sample 2 measured using NSOM. The opening diameter of the microcave is 46 µm and the depth is 175 nm. Such etching depth is deep enough to remove the entire p-GaN, p-AlGaN layer and some of the MQWs. The scanning dimension is 70 × 70 µm^2^. The MQWs PL intensity (the sum of MQWs-1 and MQWs-2) of one single microcave structure was represented by the photomultiplier (PMT) signal of the NSOM. In Figure 3a,c the outer edge of the microcave was marked with a white dashed circle. The tiny white dot-like pattern in the background of Figure 3a shows the light deflection caused by the PSS, which can be seen more clearly in Figure 3b. The small variation of 10% of the PL intensity with the period of 2.68 μm matches the period of the cone shape pattern of the sapphire substrate. The dark area inside the microcave indicates the area where the MQWs were removed. Both Figure 3a,b shows that no change of the PL intensity was found in the region 5 μm inward from the edge of the microcave. It implies that the PL does not change much for the removal of 102.6 nm epilayer. Beyond this point, the PL decrease sharply due to the removal of the MQWs. Figure 3c shows that for the Ag NPs sample, the PL intensity shows a ring-like pattern with an inner diameter of 35.2 μm and outer diameter of 46 μm. The PL intensity both inside and outside the ring is very weak compare to that of the ring. Figure 3d shows that no change of the PL intensity in the region of 1.9 μm inward from the edge of the microcave. Then it starts to increase sharply and reaches the maximum enhancement factor of 8.9 at etch depth of 75.5 nm. It is reported that LSPs can increase the density of states and the spontaneous emission rate dramatically in the semiconductor, and lead to the enhancement of light emission by LSP-QW coupling [22]. Beyond this point, the enhancement factor decreases gradually due to the emission quenching [23] and the QWs removal near the center of the ring causes further weakening of the PL intensity.

**Figure 4:**
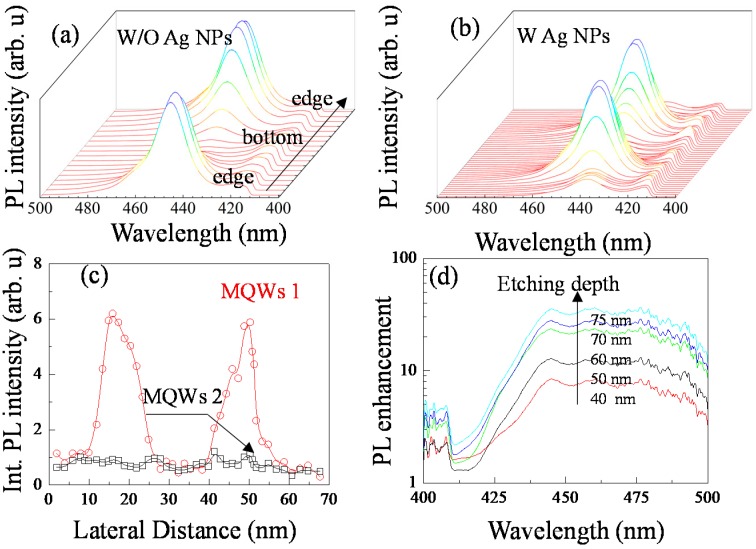
PL spectra at different positions from the outer edge of (**a**) sample 1 and (**b**) sample 2 to the bottom of the microcave. (**c**) The integrated PL intensity of MQWs-1 and MQWs-2 at different position of sample 2. (**d**) The relationship between PL enhancement factor and wavelength at different etching distance of sample 2.

To differentiate the SP enhancement between MQWs-1 and MQWs-2, PL spectra at different locations along the microcave of sample 1 and 2 were measured as shown in Figure 4a,b. The integrated PL intensities for each set of MQWs were shown in Figure 4c. The dependency of the PL intensity of MQWs-1 on the etching depth follows the trend shown in Figure 3b,d. But the PL intensity of MQWs-2 stays almost constant and is independent of the etching depth. Figure 4d shows the calculated enhancement ratio as a function of wavelength at 5 different etching depths varying from 40 to 75 nm. At etching depth of 75 nm, the local maximum enhancement is 19 when the wavelength is greater than 445.2 nm, and began to decrease after 479.5 nm. At the shorter wavelength side of 445.2 nm, the enhancement monotonically decreases, and the factor range measured at 417 nm is much smaller: 1.7–2.6. Several other microcavities were measured, and the MQW-2 of some of them showed no enhancement. With increased etching distance the electric field intensity of local surface plasmas increases rapidly. The LSP-MQWs coupling improves the spontaneous energy transfer from the MQWs electron-hole pairs to the electromagnetic localized surface-plasmon modes of the Ag NPs and suppress the non-radiative recombination. But MQWs-2 seems not to be much involved. There are two reasons behind such enhancement selectivity. One is that the physical distance between MQWs-2 and Ag NPs exceeds penetration depth of the LSP. The other reason is that the peak wavelength of MQWs-2 is far away from the center of the extinction curve.

**Figure 5:**
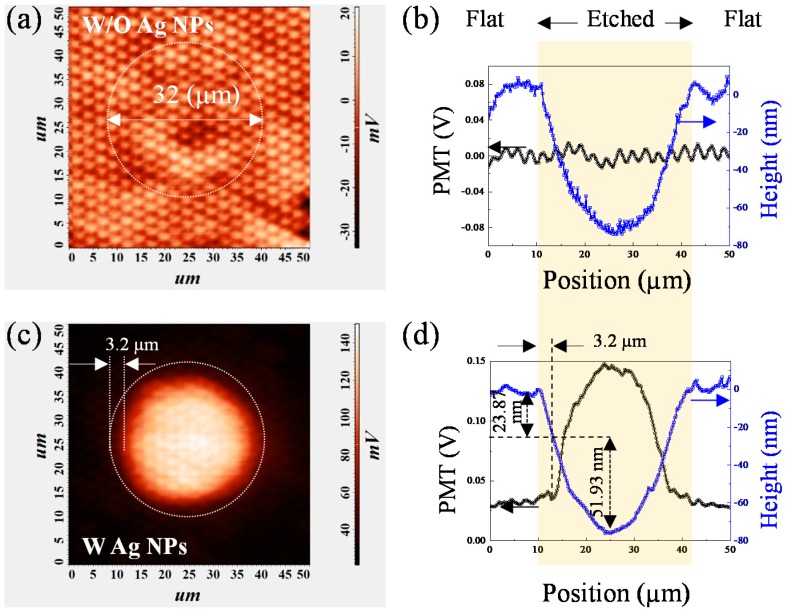
(**a**) The near-field PL mapping and (**b**) the PL and height profiles of cross sections through the center of the microcave of sample 3 without Ag NPs; (**c**) The near-field PL mapping and (**d**) the PL and height profiles of cross sections through the center of the microcave of sample 4 with Ag NPs.

With such knowledge, the second group of samples with etching depth of 75 nm was prepared. Figure 5a,b show the PL mapping and cross-sectional intensity profile of sample 3 (without Ag NPs) and sample 4 (with Ag NPs) at 1.30 MW/cm^2^. The opening diameter is 32 µm and the actual depth is 75.7 nm, measured by NSOM. According to our calculation, all the MQWs were intact with such etching depth. Due to the smaller scanning size of 50 × 50 μm, the PSS is more clearly seen than sample 1. Once again, the small variation of 10% of PL intensity with the period of 2.68 μm matches the period of the PSS. Other than that there seems no correlation between the etching depth and the PL intensity pattern. This shows that the aspect ratio of the microcave is so large that the change of LEE caused by its surface morphology is even smaller than that of the PSS substrate. Different from Figure 3a, there is no dark circle in sample 3 because the MQWs were not etched. A bright circular pattern, rather than a ring pattern, was found in sample 4 with Ag NPs. The PMT signal intensity stays almost the same for the first 23.8 nm removal of the p-GaN, which corresponds no change of PL intensity mapping for the region 5 μm inward from the edge of the microcave (Figure 5c). Beyond that, the PL intensity increase sharply for the next 51.9 nm. And the maximum PL intensity at the bottom is about 7.5 times that outside the microcave (Figure 5d).

**Figure 6:**
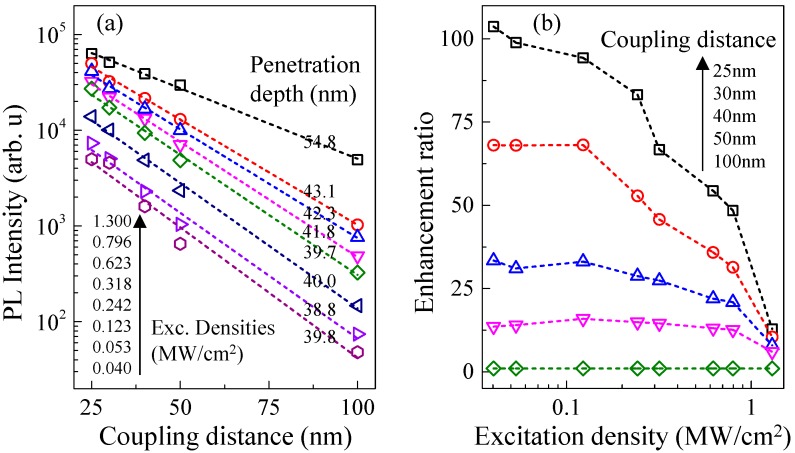
(**a**) The excitation power dependent near-field PL vs. position of Ag NPs sample 4 (**b**) The corresponding enhancement vs. excitation power of Ag NPS sample 4 at different coupling distances.

**Figure 7:**
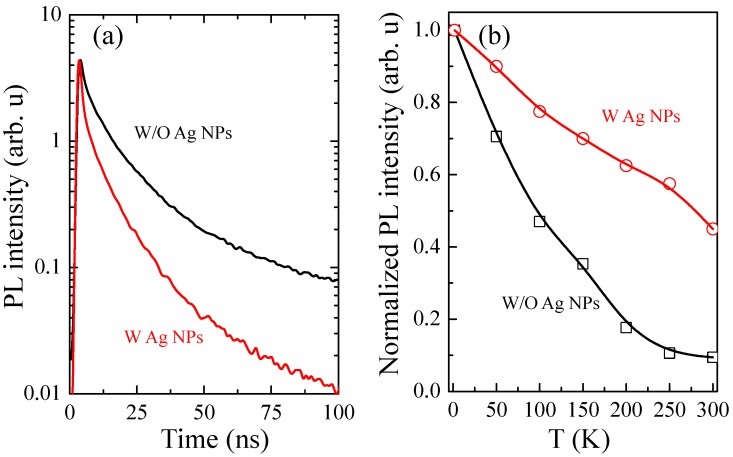
(**a**) Room temperature time-resolved PL (TRPL) and (**b**) Temperature dependent PL measurement of Ag NPs sample 4 and sample 3 without Ag NPs, respectively.

In addition to the coupling distance, the enhancement factor is actually related to the excitation power. Figure 6a shows near-field excitation power dependent peak PL intensity of MQWs-1 as a function of the coupling distance for sample 4. It shows that the PL intensity and the enhancement factor increase exponentially with the etching depth. It matches the model of SP-QW coupling: the SP is an evanescent wave and its field intensity exponentially decays with distance from the metal surface 6. Beyond this distance, the SP electric field decreases rapidly and the coupling efficiency decreases. It increases slowly from 39.8 nm at 0.040 MW/cm^2^ to 43.1 nm at 0.796 MW/cm^2^. Then it suddenly jumps to 54.8 nm at 1.3 MW/cm^2^. In Figure 6b we obtained the enhancement ratio as a function of the excitation power densities under different coupling distance. At large coupling distance, such as 100 nm, the enhancement along the excitation power has little change. At small coupling distance, the enhancement factor increases rapidly with the decrease of power densities. At a minimum coupling distance of 25 nm, a maximum enhancement of 103-fold was measured at a minimum excitation density of 0.040 MW/cm^2^. At this power density, enhancement factor of 13.6, 32.4, and 68.0 were measured at the coupling distance of 50, 40, and 30 nm, respectively. All though the penetration depth was enhanced at large excitation density, the enhancement ratio was reduced. This is consistent with the inverse relationship between the theoretically expected IQE enhancement and the initial IQE without Ag NPs [19,24]. With the increase of excitation power, the density of state of LSP becomes saturated. The polarization-induced electric field will be weakened due to the increased free carrier screening effect [17]. Therefore, exciton dipole coupling with LSP is reduced and the contribution of LSP coupling effect to radiation recombination is relatively small [25,26]. Room temperature time-resolved PL (TRPL) of samples with and without Ag NPs was measured respectively (Figure 7a). The PL decay time of each sample can be obtained by fitting the decay slope of the early decay on the semi-log profile. Under this specific excitation power, the PL decay time is 5.86 ns for the sample 3 without Ag NPs and 1.47 ns for sample 4 with Ag NPs, which indicates that the radiation recombination rate is faster due to the induced SP coupling. Figure 7b shows the normalized PL intensity as a function of different temperatures of samples with and without Ag NPs. The IQE was estimated as PL_@300K_/PL_@2K_ under the assumption that at low temperatures (typically 2–10 K) the contribution of non-radiative recombination is very small and IQE ~100%. The IQE of sample with Ag NPs was found increased by a factor of 4.7. QW-SP coupling can suppress the non-radiative recombination channels and then improve IQE due to the fast energy-transfer from the QWs to SPs, which can be also evidenced by the decrease of PL decay time of TRPL. The energy transfer efficiency from standard recombination to the LSP mode, so called Purcell Factor Fsp was calculated to be 4 [5,26]. Such Purcell Factor represents the whole sample at this particular excitation condition due to the large laser spot coverage and was an average value regardless the coupling distance.

## 4. Conclusions

In this study, we clearly observed the gradual evolution of SP coupling effect with the change of coupling distance in nanometer scale of a pseudo micro-LED. At low excitation density, the PL intensity of Ag NPs coated microcavity array blue LED is increased by 103 times. The selective enhancement of dual-wavelength MQWs is partially due to the overlapping of emission spectrum and extinction curves and is partially affected by the coupling distance between LSP and MQWs. The penetration depth of LSP effect was experimentally measured between 39–55 nm in our samples depending on the excitation density. Even though the penetration depth of the SP effect was the largest at the highest excitation power density, the enhancement factor is the lowest because of the saturation of the density state of LSP. Our results provide a guideline for the design and fabrication of high-efficiency micro-LEDs.
